# A Universal Pedicle Screw and V-Rod System for Lumbar Isthmic Spondylolysis: A Retrospective Analysis of 21 Cases

**DOI:** 10.1371/journal.pone.0063713

**Published:** 2013-05-17

**Authors:** Xiong-sheng Chen, Sheng-yuan Zhou, Lian-shun Jia, Xiao-min Gu, Lei Fang, Wei Zhu

**Affiliations:** Department of Orthopedics, Shanghai Changzheng Hospital, Shanghai, China; Universität München, Germany

## Abstract

**Objective:**

To investigate the surgical outcome of a universal pedicle screw-V rod system and isthmic bone grafting for isthmic spondylolysis.

**Methods:**

Twenty-four patients with isthmic spondylolysis at L5 and grade 0–I spondylolisthesis (Meyerding classification) received isthmic bone graft and stabilization using the universal pedicle screw-V rod system. Back pain was evaluated using the visual analog scale (VAS) and time to bone healing, improvement in spondylolisthesis and intervertebral space height at L5/S1 and L4/L5 were assessed.

**Results:**

Twenty-one patients were followed up for 24 months and included in the analysis. Back pain was markedly improved at 3 months postoperatively with a statistical difference in VAS scores compared with preoperative VAS scores (*P*<0.001). The VAS scores were 0 to 3 at 6 months postoperatively in all patients and no back pain was reported in all patients except 2 patients who complained of back pain after prolonged sitting. X-ray examination showed a bone graft healing time of 3 to 12 months. Grade I spondylolisthesis improved to grade 0 in 4 patients and no noticeable change was observed in the remaining 17 cases. The intervertebral space height at L5/S1 was statistically increased (P<0.05) while no statistically significant change was seen at L4/L5. There was no statistically significant difference in the ROM of the intervertebral disks of L5/S1 and L4/5 before and after surgery.

**Conclusions:**

The universal pedicle screw-V rod system and isthmic bone grafting directly repairs isthmic spondylolysis and reduces back pain, prevents anterior displacement of the diseased segment and maintains intervertebral space height, thus offering a promising alternative to current approaches for isthmic spondylolysis.

## Introduction

Lumbar isthmic spondylolysis is commonly seen in adolescents [1]–[3] and often occurs at L5 [4], [5]. Conservative therapy is recommended for adolescents with initial onset of the disease [6] while surgery is considered after conservative therapy has failed, the disease becomes aggravated, or lumbar spondylolisthesis has progressed [7]–[9]. Currently, surgical methods include single vertebra body direct repair, single or multiple segment fixation and isthmic repair and fusion. Single or multiple segment fixation refers to intervertebral body fusion involving one or at least one intervertebral space. Although 75% to 100% of patients show improvement after intervertebral body fusion, compensatory activities of adjacent vertebral segments are increased, which increases the risk of a second surgery due to diseases in the adjacent intervertebral space [10], [11].

Direct single vertebra body repair is aimed at the placement of a stabilization device in the diseased vertebra to ensure proper healing of bone graft in the isthmus. The surgery, which is favored by some orthopedic surgeons, does not increases burden on adjacent intervertebral spaces no intervertebral body fusion is carried out [12]–[14]. Due to the unique anatomic features of bone structure adjacent to lumbar isthmic spondylolysis, proper internal stabilization method for lumbar isthmic spondylolysis has been intensely debated. A myriad of stabilization methods have been reported, including isthmic Buck screw fusion [15], stabilization of the diseased vertebra by wiring between the spinous process and the transverse process [16], stabilization by the combined use of pedicle screw and wiring or cable [17], stabilization by the joint use of pedicle screw and a hook screw [18], [19], or stabilization with pedicle screw-V rod [20], [21]. These methods have achieved variable success, but are hampered by their intrinsic defects. In Buck screw fusion surgery, as the screw directly passes through the isthmus, which noticeably reduces the amount of bone graft in the isthmus, bone healing is compromised. Stabilization with wiring and titanium cable requires the use of lumbar brace or prolonged immobilization. The pedicle screw-vertebral plate hook system and the pedicle screw-V rod system offer better stabilization than the other methods [19]. However, the pedicle screw-vertebral plate hook system is complicated and, when the vertebral plate hook is improperly positioned, injury to the dural sac or nerves ensues.

The pedicle screw-V rod system was first reported by Gillet *et al*. in 1999 [20]. The authors used single axis pedicle screw and a V rod that has a sagittal upper curve to fit the vertebral plate. The single segment pedicle screw-V rod is safer than the pedicle screw-vertebral plate hook system, but bending and placement of V rod is technically difficult. To ease the technical difficulty involved in the use of the pedicle screw-V rod system, we designed a multiple axis pedicle screw with an adjustable end that facilitated the assembly of V rod. In this study, we retrospectively analyzed the surgical outcome of 21 patients with bilateral isthmic spondylolysis who were treated with the modified pedicle screw-V rod system at our institution. We found that the modified pedicle screw-V rod system can effectively alleviate the symptoms of patients with lumbar isthmic spondylolysis and prevent the anterior slipping of the diseased vertebra and improve or maintain intervertebral space height.

## Subjects and Methods

### Subjects

We retrospectively reviewed the surgical and radiological data ofpatients with bilateral isthmic spondylolysis at L5 who were treated by one of the surgery groups at Shanghai ChangzhengHospitalbetween Dec 2004 to Jan 2009. X-ray and CT evaluation of spondylolysis and spondylolisthesis was performed as previously described [21], [22]. The presence of spondylolisthesis at a spinal segment with bilateral spondylolysis was considered isthmic spondylolisthesis while the presence of spondylolisthesis in the absence of spondylolysis was considered degenerative spondylolisthesis. Major inclusion criteria were 1) patients with isthmic spondylolisthesis of 0 and Igrades and levels with low back pain with or without sciatica, 2) severe back pain that compromised the patient’s quality of life despite conservative therapy for at least three months. 3) Grade I to II lumbar degenerative disk changes atL5/S1 [25]. A patient was excluded if he or she had osteoporosis, diabetes mellitus or any other diseases that could interfere with bone fusion, or if he or she had previous spine surgery, psychiatric disorders and drug or alcohol abuse.

### Surgical Procedure

**Figure 1:**
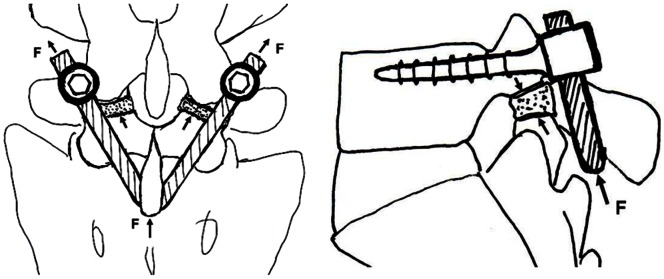
Vertebral stabilization by the pedicle screw-V rod system. Force F (arrow indicates direction of F) compresses the isthmic bone graft through the V rod, the spinous process and the vertebral plate. Meanwhile, the pedicle screw-V rod system prevents anterior displacement of the diseased segment and does not affect flexion and axial rotation of the spine.

The supraspinal ligaments were preserved during surgical exposure and a universal pedicle screw was inserted into the pedicle on each side at L5. Approximately 1/5 (2 to 3 mm) of the articular process under L4 was excised with an osteotome, with care taken to remove as little capsular structure as possible, for exposure of lesions in the isthmus of L5. Scar tissues were excised down to bleeding bone. Bone graft was harvested from the right iliac crest and was trimmed into 2 wedged bone blocks to be placed in the defect in the isthmus, and the adjacent area was filled with morselizedcancellous bone. A titanium rod was bent in a “V” shape and put under the L5 spinous process after removal of the L5–S1 interspinous ligament. The rod was firmly pushed anterosuperiorly to lock onto the pedicle screw so that the V-shaped rod, the spinous process and vertebral plate jointly promoted the compression of the bone graft in the defect and stabilized the posterior arch (Fig. 1). In patients with nerve root canal stenosis, a small portion of the distal aspect of the lamina, where stenosis occurred, was excised and the yellow ligament was also excised and the nerve root canal was dilated to achieve decompression. The proper positioning of the implants was checked by intraoperative radiography.

### Ethics Statements

The study protocol was approved by the ethics review board of Shanghai Changzheng Hospital, Shanghai. We have obtained written informed consent from all study participants. All of the procedures were done in accordance with the Declaration of Helsinki and relevant policies in China.

### Clinical Evaluation

Lumbar spondylolisthesis was graded according to the Meyerding classification. [23] Patients were followed up at 3, 6, 12 and 24 months at the outpatient clinic and once every year thereafter. Back pain was evaluated using the visual analog scale (VAS) where 0–3 indicates mild pain, which is tolerable and does not interfere with rest; 4–6 represents pain that interferes with rest, but is still tolerable; 7–10 indicates intolerable pain. VAS scores were collected from patients preoperatively, postoperatively and at the last follow-up.

### Radiological Evaluation

**Figure 2:**
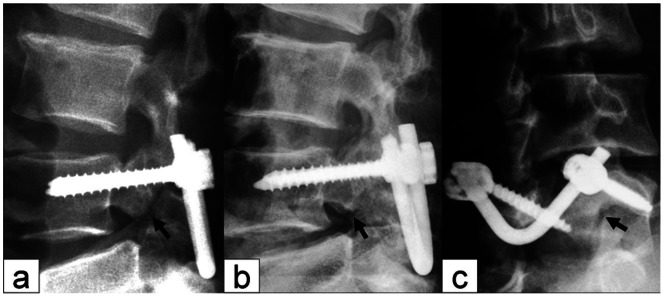
X-ray manifestations of isthmic bone graft healing. (a) The isthmic bone graft was partially healed; (b) the isthmic bone graft was fully healed; (c) the oblique view shows healing of the isthmus; no “dog neck sign” was observed. Arrow indicates the isthmus.

**Figure 3:**
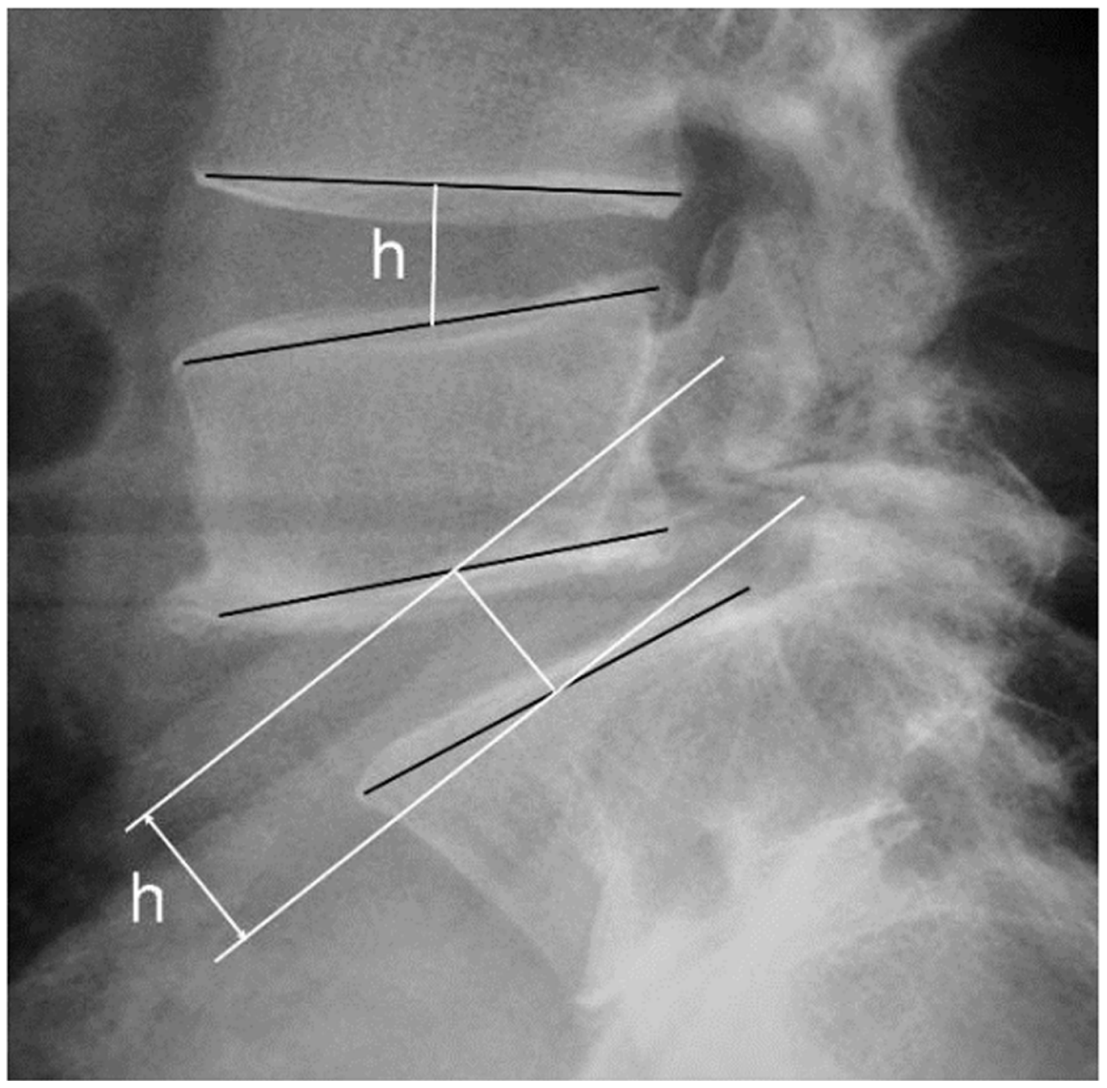
Measurement of intervertebral space height. The intervertebral space height (h) is determined by the distance from the midpoint of the superior endplate to the midpoint of the inferior endplate.

Preoperative radiological evaluation included anteroposterior, lateral, oblique, and flexion-extension plain radiograph and CT scans. The scan data were evaluated independently by two radiologists with respect to clinical and personal data. The entire lumbar spine was reviewed using bone windows and both axial views and multiplanar reconstruction were analyzed.Anteroposterior, lateral, oblique, and flexion-extension plain radiographs were evaluated for healing of isthmic bone graft, intervertebral space height, and spondylolisthesis at 3 d and 3, 6, 12 and 24 months postoperatively. Fusion rates were assessed by an independent radiologist at 6 months. Definitive fusion was considered to be established on static and dynamic plain X-rays by 1) the formation of trabecular bony bridges between contiguous isthmuses; 2) the interface between the preoperative low density area and bone graft shadow, and the “dog neck sign” became indistinct (Fig. 2). Fusion of bone graft was confirmed by cross-section bone windows on CT scan. 3) Intervertebral space height was measured from the mid points of the upper and lower endplate on lateral radiographs of the lumbar spine (Fig. 3). 4) The range of motion (ROM) of the angle between intervertebralspaces was the difference in the degree of angle at extension and flexion. Changes of ROM at L5/S1 and L4/5 were evaluated preoperatively and 24 months postoperatively.

### Statistical Analysis

Data were analyzed using the SPSS statistical analysis software version 13.0 (SPSS, Inc., Chicago, IL). Preoperative and postoperative VAS scores were compared using LSD-t test and the grade of spondylolisthesis prior to and 24 months following surgery was analyzed using χ^2^ test. Changes in intervertebral space height and ROM of L5/S1 and L4/L5 were compared using paired sample *t* test.

## Results

### Patient Baseline Demographic and Disease Characteristics

The clinical data of 92 patients with bilateral isthmic spondylolysis at L5 were reviewed and 68 patients were excluded. Twenty four patients with bilateral isthmic spondylolysis at L5 were eligible for the study. Patient demographic and baseline characteristics are shown in Table 1. They included 19 males and 2 females and their age ranged from 14 to 58 years with a mean age of 34.56 years. Four cases were less than 18 years of age. The VAS scores ranged from 5 to 9, with a mean of 7.5. Intermittent radiating lower leg pain or numbness was present in 7 patients, all of whom had gradeIspondylolisthesis. CT and MRI revealed nerve root canal stenosis at the involved segments in these patients. The course of isthmic spondylolysis was 7 to 180 months with a mean duration of 44.56 months. The fibrous annulus at L4/L5 was intact in all patients and no herniated lumbar disk was noted.

**Table 1:** Patient demographic and baseline characteristic.

Sex	Age (years)	BW (kg)	DDD grade	Spondylolisthesis		VAS score					
				Pre-op	Post-op24 months	Pre-op	Post-op (months)				
							3	6	12	24	Final follow-up
M	32	67	II	I	I	5	3	0	0	0	1
M	53	75	II	I	I	9	4	0	0	0	0
F	44	52	II	0	0	9	3	0	0	0	0
M	38	72	II	0	0	6	3	3	0	0	0
M	34	63	II	I	0	8	3	2	0	1	0
M	55	65	II	I	I	9	3	3	1	0	0
M	26	76	II	0	0	9	2	2	2	1	0
M	48	72	II	I	I	5	3	2	1	0	0
M	52	81	II	I	I	8	3	2	0	0	0
M	36	62	II	I	I	7	2	2	1	0	0
M	14	51	II	I	0	6	3	2	2	0	0
M	21	56	II	0	0	9	5	0	1	0	0
M	17	52	I	I	0	7	3	3	1	0	0
F	36	47	II	0	0	9	5	2	1	1	1
M	17	43	I	0	0	5	3	2	2	1	0
F	17	45	I	I	I	9	2	2	1	1	0
M	43	72	II	0	0	6	3	0	1	0	0
M	49	71	II	I	I	9	3	3	0	0	1
M	40	73	II	I	0	9	2	0	0	0	0
M	22	82	II	0	0	6	3	0	0	0	0
M	42	64	II	0	0	8	3	0	1	0	1

BW, body weight; DDD, degenerative disk disease; F, female; M, male;pre-op,preoperative; post-op, postoperative; VAS, visual analog scale;

### Surgical Outcome

Domelike decompression of the lumbar vertebral canal was performed in 7 patients who had intermittent radiating pain or numbness of the lower leg. In 3 instances, local thickening of the yellow ligament was observed and an undercutting facetectomy of the affected area was performed, and patency of the spinal canal was examined. In the other 4 instances, no bone or ligament thickening was noticed and routine decompressive surgery was performed. The mean volume of bleeding was 186 mL (range, 100 to 250 mL) and the mean drainage volume was 65 mL (range, 50 to100 mL). No patient required blood transfusion. Mild tolerable pain was reported, which required no morphine pain medicine. There was no nerve injury, wound infection or other complications. The mean duration of hospital stay was 7 days.

### Follow Up

**Figure 4:**
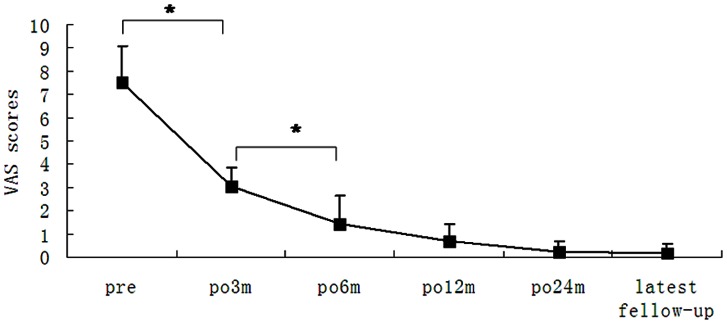
Preoperative and postoperative VAS scores. *P<0.05.

The patients were followed up for a mean duration of 35 months (range, 3 to 84 months). Twenty-one patients were followed up for more than 24 month including 24 months in 7 cases, 36 months in 5 cases, 48 months in 5 cases, 60 and 72 months each in 1 case and 84 months in 2 cases. The mean preoperative VAS score at 3 months was 7.5 (range, 5 to 9) and the mean postoperative VAS score was 3.04 (range, 2 to 5). There was a significant improvement in back pain (*P*<0.05) (Fig. 4). Intermittent radiating pain or numbness of the lower leg was effectively improved in the 7 patients. The mean VAS score at 6 months postoperatively was 1.43 (range, 0 to 3) and 1 at 24 months for 5 patients with the remaining patients pain-free. At the last follow up, the mean VAS score was 1 in 4 patients with the remaining patients pain-free. There was a statistically significant difference between the preoperative VAS score and the postoperative VAS score at 3 or 6 months (*P*<0.05).

### Radiological Outcomes

**Figure 5:**
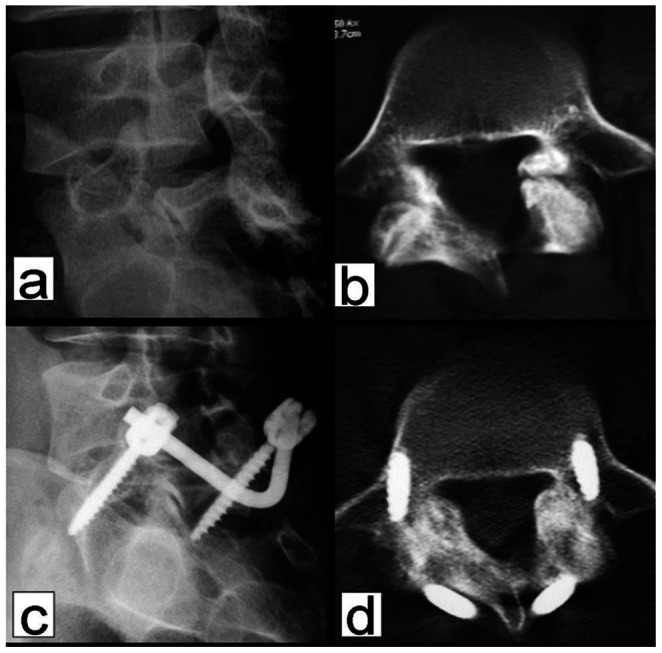
A 21-year old male patient with isthmic spondylolysis for 7 months failed conservative therapy. Preoperative oblique x-ray and CT scan using bone windows reveal isthmic spondylolysis at L5 (a and b). At 6 months postoperatively, the oblique view shows an indistinct interface between the isthmus and bone graft with increased density (c). CT scan on bone windows shows healing of the isthmic bone graft (d).

**Figure 6:**
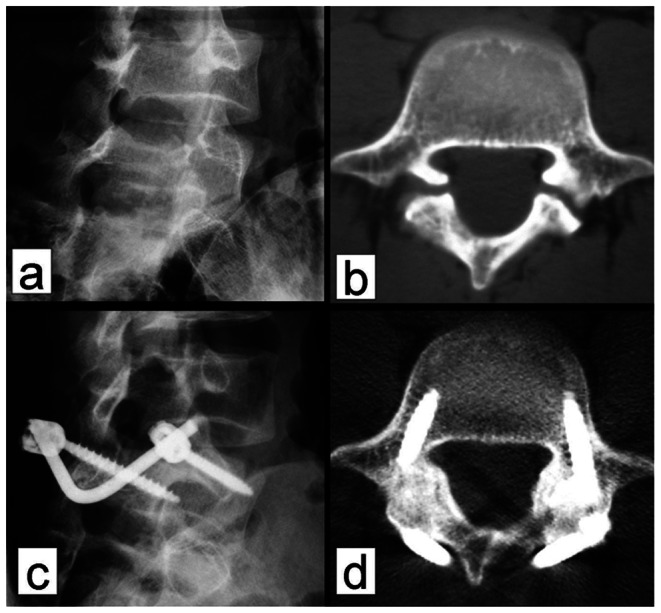
A 22-year old male patient with isthmic spondylolysis for 2 years failed conservative therapy. Preoperative oblique x-ray and CT scan using bone windows reveal isthmic spondylolysis at L5 (a and b). At 6 months postoperatively, the oblique view shows an indistinct interface between the isthmus and bone graft with increased density (c). CT scan on bone windows shows healing of the isthmic bone graft (d).

**Figure 7:**
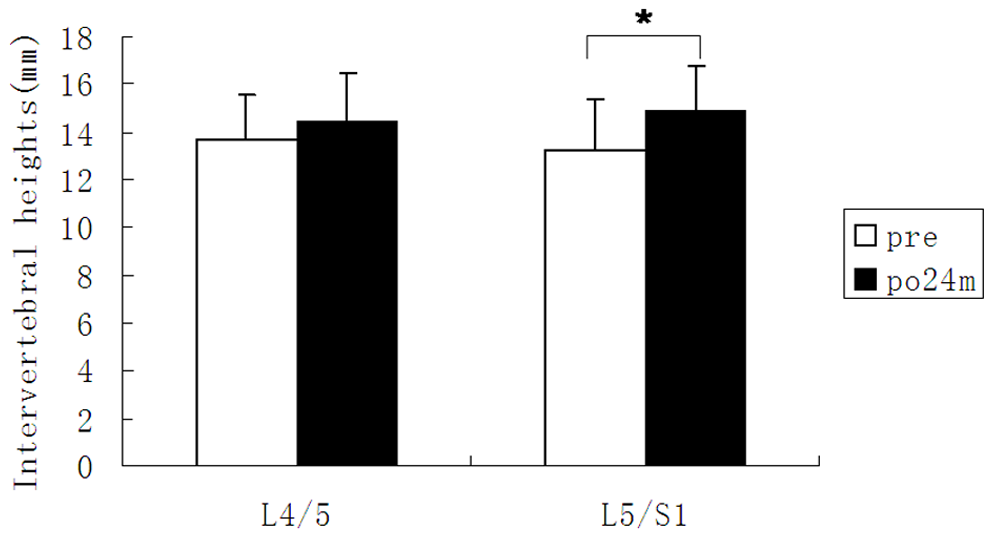
Preoperative and postoperative intervertebral space heights of 21 patietns with isthmic spondylolysis. *P<0.05.

**Figure 8:**
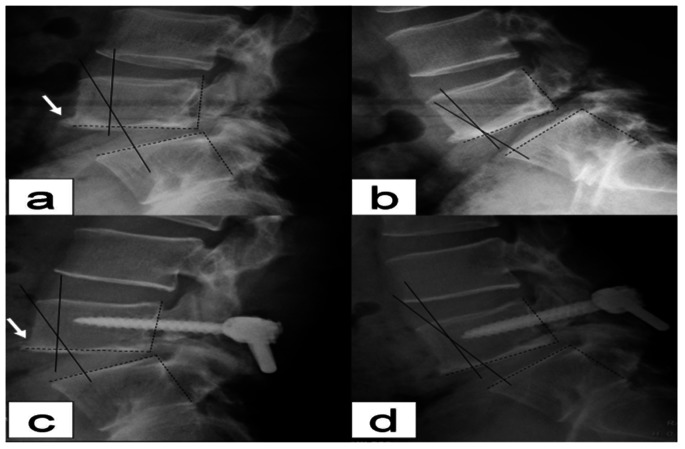
a. Spondylolisthesis of L5/S1 at 0 grade overextension prior to surgery. b. Spondylolisthesisof L5/S1 at I gradeoverflexion, indicating lack of dynamic stabilization. c. Displacement of L5/S1 at 0 gradelateral overextension 4 years post surgery. d. Displacement of L5/S1 at 0 gradeoverflexionwith no apparent limitation of range of motion of the intervertebral disk of L5/S1 4 years post surgery. The bony outgrowth at the anterior inferior edge of L5 (indicated by arrow) becomes smaller at 4 years postoperatively (c) compared with preoperative image (a, arrow), suggesting that L5 and S1 achieved good stabilization following surgery.

Static and dynamic plain X-ray examination showed that the mean duration of bone fusion was 6 months (range, 3 to 12 months) (Fig. 5 and 6 and Table 2). Bone fusion was confirmed by CT scan in 6 cases, who all showed excellent bone fusion. Measurement of the intervertebral space height in 21 patients with isthmic spondylolysis revealed that the preoperative intervertebral space height at L5/S1 was 13.2±2.1 mm, which was significantly lower than that at 24 months post operation (*P*<0.05) (Fig. 7). There was no statistical difference between the preoperative intervertebral space height (13.7±1.9 mm) and the postoperative intervertebral space height at L4/L5 (14.4±2.2 mm) (*P*>0.05). Seven patients showed increased displacement upon overflexion or overextension prior to the operation, which was effectively corrected postoperatively. One of the patients showed bony outgrowth at the anterior inferior edge of L5 before the surgery, which was apparently absorbed 4 years post operation (Fig. 8).At 24 months of follow up, 4 patients who had preoperative grade I spondylolisthesis improved to grade 0 spondylolisthesis while in the remaining 17 patients there was no change in the grade of spondylolisthesis (Table 3). There was no statistical difference in the ROM of L5/S1 and L4/5 intervertebral space before and after surgery (*P*>0.05) (Table 4).

**Table 2:** Time to bone graft healing of 21 patients with isthmic spondylolysis.

Bone graft healing time (months)	3	6	9	12
No. of cases	9	5	5	2

**Table 3:** Preoperative and postoperative grade of spondylolisthesis in 21 patients with isthmic spondylolysis.

	Grade 0	Grade I
Preoperative	9	12
Postoperative (24 months)	13	8

χ^2^ test, *P* = 0.149.

**Table 4:** The range of motion (ROM) of L5/S1 and L4/5 before and 24 months after surgery.

ROM	Preoperative	24 months post operation
LS/S1*	9.4±2.0	9.3±1.6
L4/5^#^	8.9±1.7	9.3±2.1

* and ^#^, P>0.05.

## Discussion

In the current study, we treated 21 patients with isthmic spondylolysis with a universal pedicle screw-V rod system and found that the VAS scores were significantly reduced at 3 months postoperatively. X ray examination further revealed that the isthmic bone graft can achieve bone fusion after 3 to 12 months. Isthmic bone graft healing is not coincident with back pain reduction. In eight patients who were pain-free, only five patients showed bone healing at 6 months postoperatively. All patients had a VAS score <3 at 6 months postoperatively, but bone healing was noticed in only 14 patients (14/21) on x-ray examination. These observations suggest that bone graft healing is not a sole contributor to back pain alleviation. Vertebral stabilization by the pedicle screw-V rod system may offer additional contribution to back pain relief. These findings are consistent with those reported by Koptan *et* *al.* [24]. In addition, no patients showed worsening of spondylolisthesis during the follow up. These findings indicate that the universal pedicle screw-V rod system can effectively prevent anterior displacement of the diseased vertebral segment.

Sairyo *et al.* [13] showed that direct repair of the isthmus could improve the biomechanical environment of the diseased and adjacent intervertebral disks.The pedicle screw-V rod system directly repairs the isthmus of the vertebra and the procedure does not impact on adjacentvertebral segments and causes noinjury to thediseased intervertebral disc. Our findings indicate that intervertebral space height (L5/S1) markedly improved at 24 months postoperatively. No anterior nor posterior displacement at L5 was observed in all patients upon overflexion or overextension, indicating that the pedicle screw-rod system can effectively prevent anterior displacement of the diseased segment. The screw-V rod could prevent anterior displacement of L5 vertebra (Fig. 1), thus lessening the forward shear stresson the intervertebral disk ofL5/S upon flexion. The V rod is indirectly placed above the sacral crest (Fig. 1), which partially transmits the vertical load on the intervertebral disk of L5/S1 to the sacral crest through the pedicle screw-V rod system. This could partially lesson the vertical load on the intervertebral disk of L5/S1. In addition, there was no marked difference in the ROM of the intervertebral disk ofL5/S1 before and following surgery, suggesting that the pedicle screw-V rod system does not impact the motion of the intervertebral disk of L5/S1 while stabilizing the vertebral segment. In addition, there was no significant change in the height and ROM of the intervertebral disk ofL4/5 prior to and after surgery, indicating that there is no increased burden on the cephalic adjacent segment (L4/5). Thus, the modified pedicle screw-V rod system provides elasticity in stabilization, exerting no apparent effect on flexion and extension of the spine while preventing anterior displacement of the diseased vertebral segment. Our three dimensional finite element analysis of the modified pedicle screw-V rod system for stabilizing the bilateral isthmus of L5 showed that the system applied pressure axially and not only provided good stabilization for anterior flexion and back extension, lateral flexion and rotation but also maximally preservedthe motion of the adjacent vertebral segments (manuscript in preparation). It was particularly effective in stabilizing the caudal vertebral segment during rotation. In one patient, roentgenography at 4 years of follow up revealed apparent absorption of bony outgrowth at the anterior edge of L5 vertebra (Fig. 8), suggesting effective stabilization of L5-S1 by the modified pedicle screw-V rod system. As a result, these properties of the pedicle screw-V rod system offer a more favorable kinetic environment for the intervertebral disk, which facilitates the improvement of the intervertebral disk.

**Figure 9:**
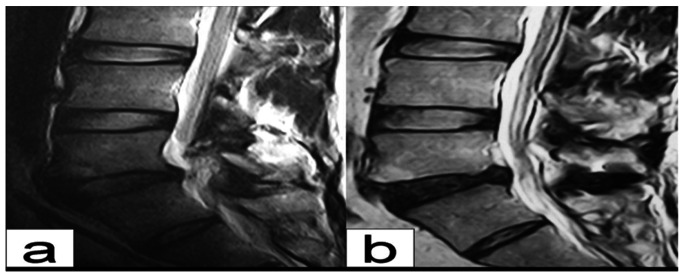
A 39 year old male patient with isthmic spondylolysis at L5. a grade II disk degeneration of L5/S1 before surgery; b grade III disk degeneration was noted at L5/S1 4 years post operation. No change in vertebral height was noted and the endplate was smooth. The intervertebral disk of L4/5 showed no apparent changes compared with preoperative findings.

Gillet *et* *al.* [20] did not recommend the pedicle screw-V rod system for patients with degenerative disk disease and spondylolisthesis while Koptan *et* *al.* [24] reported satisfactory outcomes in isthmic spondylolysis patients with grade I spondylolisthesis but no degenerative disk disease. Our results indicate that the universal pedicle screw-V rod system can improve the loading environment of intervertebral disks and mild degenerative disks may cease degeneration or even improve with enhancement of the loading environment. The method is applicable to isthmic spondylolysis patients with grade I spondylolisthesis and grade I or II degenerative disk disease with intact fibrosus annulus [25]. Lumbar MRI is not recommended for conventional use for outpatient follow up visits due to its high cost. MRI data were available in 6 patients and in one patient, though grade II L5/S1 disk degeneration before surgery changed to grade III, there was no reduction in vertebral height and no degeneration in the endplate was observed. In addition, the patient complained of no lumbar pain and the isthmus was well healed and no degenerative changes were observed at the intervertebral disk ofL4/5 post operation (Fig. 9) . In the remaining5 cases who were followed up for 1 to 3 years, MRI showed no noticeable degenerative changes at the intervertebral disks of L5/S1 and L4/5. Currently, no patient has resorted to second surgery due to lumbar disk degeneration or other causes.

In conclusion, the universal pedicle screw-V rod system directly repairs lumbar isthmic spondylolysis. It is easy to maneuver and minimally invasive. Moreover, it is effective in alleviating back pain, achieving bone graft healing, preventing anterior displacement of the diseased segment.It also does not impact on the ROM of the diseased segment and adjacent segments and it improve and maintains intervertebral space height. The modified system offers an effective alternative to current approaches for isthmic spondylolysis.
